# Electric vehicles in Bangladesh: Impact on the environment, mobility, and the economy of an impending ban

**DOI:** 10.1002/puh2.43

**Published:** 2022-12-07

**Authors:** Syeda Nurunnahar, Md Shariful Islam, Mahbubur Rahman

**Affiliations:** ^1^ Infectious Diseases Division icddr,b Dhaka Bangladesh

**Keywords:** electric vehicle, environment, economy

## Abstract

The government of Bangladesh recently decided to outlaw electric three‐wheelers due to the rise in accidents on highways. Although the restriction is intended to improve road and transportation safety, it will only exacerbate the current economic crisis if alternate sources of income are not made available. Additionally, the use of battery‐powered electric three‐wheelers is significant for providing short‐distance transportation in rural areas and the commercial centers of metropolitan cities. In many villages, electric three‐wheelers are the only accessible mode of rapid transportation. Moreover, electric vehicles are environmentally beneficial because their increased use will lower the consumption of fossil fuels and CO_2_ emissions from the transportation sector. Banning electric three‐wheelers will have serious consequences for the environment, mobility, and the economy. Instead, the government can bring existing three‐wheeled motorized vehicles under Bangladesh Road Transport Authority supervision. Monitoring and policy making are easy when a mode of transport belongs to the formal sector. Adding necessary measures to use of the vehicle will boost mobility and save Bangladesh's environment and economy.

## INTRODUCTION

Air pollution is among the environmental concerns which adversely impact human beings. In 2021, it contributed to 6.5 million deaths worldwide [1]. A major source of air pollution is the high number of conventional vehicles [2]. Such vehicles run on diesel or gasoline. As a result, they emit substances such as carbon monoxide, nitrogen oxides, hydrocarbons, and particulate matter [3]. It is these substances that cause air pollution. Air pollution then leads to health problems like asthma, bronchitis, lung cancer, emphysema, heart disease, and death [2]. Once again, Dhaka City of Bangladesh was ranked very low on the Air Quality Index, with a particulate matter (PM)2.5 reading of 83.30 μg/m^3^, which is alarming [2, 4]. Bangladeshi cities are vulnerable to air pollution due to vehicular emissions, industrial pollution, and dust accumulation from different sources [3]. Baby‐taxis (motorized rickshaws), tempos, mini‐trucks, motorcycles, and other vehicles with two‐stroke engines are the primary source of air pollution in Dhaka City, as recently observed by scientific research conducted by specialists at the Department of Environment (DoE) of Bangladesh along with other relevant organizations in the country. Currently, more than 500,000 motor vehicles, including about 65,000 baby‐taxis, are plying their trade in Dhaka City alone [4]. In addition, a high number of trucks and mini‐buses, which are overloaded, poorly maintained, and very old, are also plying their trade on the streets, emitting gases and smoke. Indeed, more than 80% of the vehicles in daily use on the streets of Dhaka City are defective, and these types of vehicles emit far greater quantities of black smoke than allowed. Vehicles using petrol and diesel emit black smoke which contains unburned fine carbon particles [2]. Non‐motorized vehicles, particularly at road intersections, are significantly responsible for the severe congestion and thus enhance emission problems. Non‐motorized transport accounts for 80% of total trips, and motorized transport accounts for only 5.9% [2].

Electric three‐wheelers are motorized vehicles powered by lead‐acid batteries and were initially a measure to help disabled people earn a living [6]. However, following their adoption by urban commuters and given their greater cost‐efficiency than other vehicles, the number of motorized three‐wheelers on roads has rapidly increased over the past ten years. Approximately 1 million electric three‐wheelers currently operate in Bangladesh, a number projected to grow to 2.5 million by 2025 (best professional estimate). Battery‐operated electric vehicles is environmentally friendly[7] as it does not emit any particular matter or CO_2_. Electric three wheelers play a significant role in short‐distance transportation services in urban and rural areas.

### The legislative and regulatory perspective on electric three‐wheelers

In a policy shift, the Bangladesh government decided to ban electric three‐wheelers, reasoning that such vehicles were responsible for an increasing number of accidents on highways. On December 15, 2021, they were banned in Bangladesh. The High Court observed that battery‐run three‐wheelers do not have a proper brake system for the rear wheels or a license from the Bangladesh Road Transport Authority (BRTA) and that unlicensed vehicles do not have permission to be on the roads.

### Vehicle mishap in Bangladesh

**FIGURE 1:**
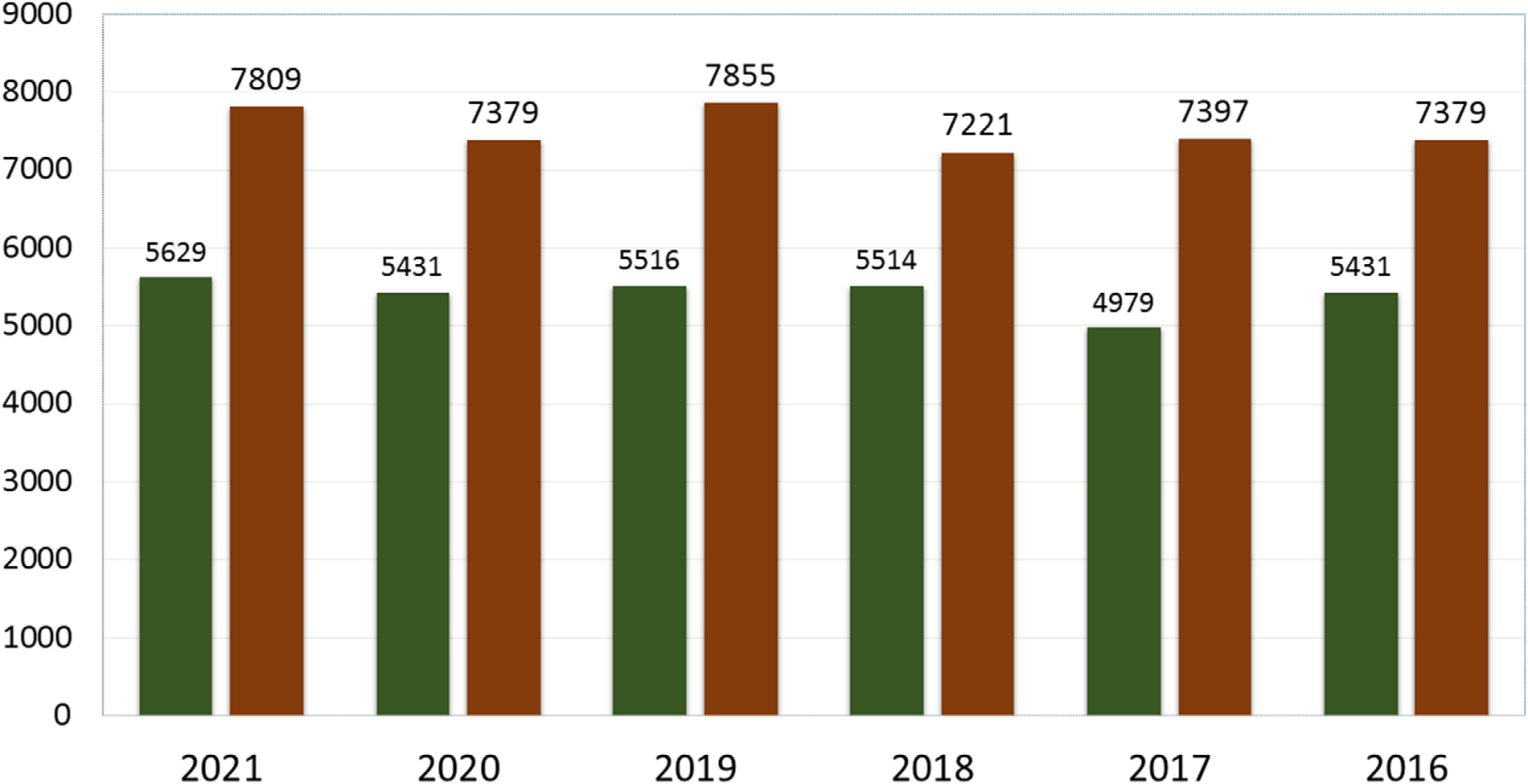
Year‐wise road crashes and deaths

**FIGURE 2:**
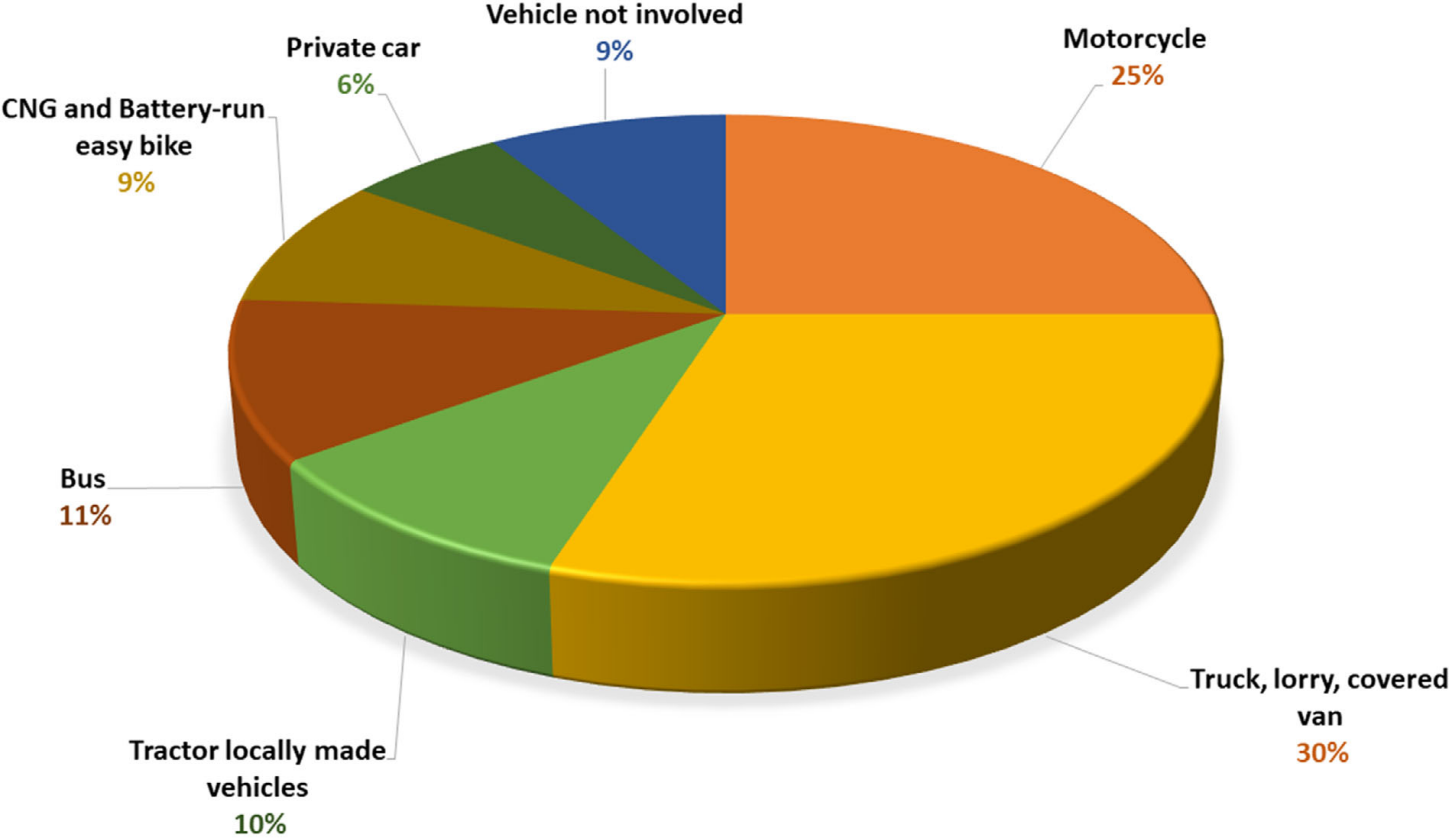
Proportion of accident by vehicle in Bangladesh 2021

**FIGURE 3:**
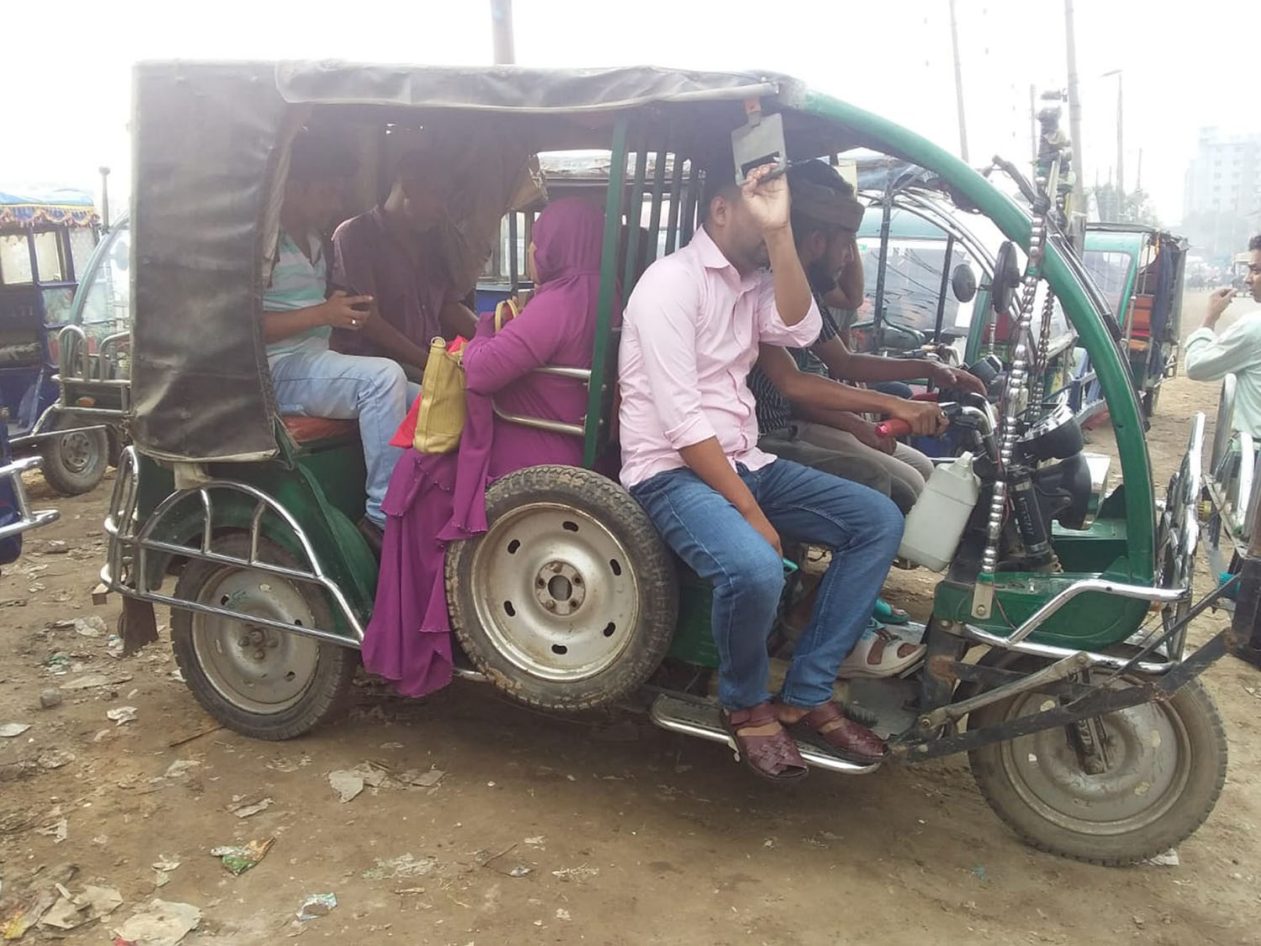
An electric three‐wheeler full of passengers

The number of road accidents is increasing every year. A report shows that in Bangladesh from 2016 to 2019 vehicles such as trucks and covered vans were responsible for 29.81% of accidents, a figure that increased by 0.19% in 2021. Motorcycles were responsible for the second highest proportion of deaths, 21.4%, which increased by 3.6% in 2021, while the rate of bus accidents decreased by 7.99%. Battery‐run easy bikes were responsible for 8.04%, a figure which has now risen to 9% (Figures 1, 2 and 3).

### Electric three‐wheelers facilitate mobility: a global consumer demand

UN Environment supports electric two and three‐wheeler projects in sixteen countries in Africa and Asia: Ethiopia, Morocco, Kenya, Rwanda, Uganda, Burundi, Madagascar, Sierra Leone, Tanzania, Philippines, Thailand, Vietnam, Bangladesh, India, Maldives, Nepal [8]. If three‐wheeler motorized vehicles go off‐road, mobility will be difficult for low to middle‐income commuters. Besides, electric three‐wheelers mostly run on narrow roads of the locality as a substitute for door‐to‐door service. United Nations Environment Program, in a study, calculated that a global shift to 90% battery‐electric motorcycles sales by 2030 could result in CO2 emissions reductions of about 11 billion tons between now and 2050 [9].

### Another wave of mass unemployment

The ban was issued for reasons of road and transport safety, but multiple economic crises will ensue if the government does not support electric vehicle drivers to find an alternate source of income. People have not overcome the financial jeopardy caused by the COVID‐19 pandemic yet. During the first and second waves of COVID‐19, transport sector workers had the highest rate of job loss. The Bangladesh Institute of Labor Study's recent findings shows that 95% of transportation workers lost their jobs between April 5, 2020 and August 10, 2021, when the government ordered countrywide restrictions to contain the spread of the infection [8]. The livelihoods of almost 1.5 million people and their family members are dependent on three‐wheelers [10]. Overcoming financial loss and finding new ways of supporting themselves is hard for the drivers as driving is their only way to earn a living. Banning and vandalizing electric three‐wheelers will create an additional burden on the already economically vulnerable drivers. Additionally, importers and local battery manufacturer companies will share the financial loss since 70% of battery demand is attributed to easy bikes and auto rickshaws [11].

### Lead intoxication from informal used lead‐acid battery recycling in Bangladesh

Following the ban, police have already *confiscated* at least 13,000 illegal electric motor‐run rickshaws and vans [12]. If a vast number of motorized vehicles is dumped without proper regulation, it will create another environmental crisis. A minimum of four 12‐volt dry lead‐acid batteries are used in one battery rickshaw. Five 12‐volt lead‐acid batteries are used in easy bikes. Such batteries are made of lead cadmium, which is carcinogenic if recycled in the open air.

Approximately half of the industry's lead supply is sourced from used lead‐acid batteries (ULABs) that are recycled by small informal enterprises in the open air which have serious consequences on health and environment [13]. There are more than 1000 battery recycling and recharging sites nationwide [14, 15].

Bangladesh is the fourth worst‐hit country in the world in terms of the number of children affected by lead poisoning, as per a recent global report by UNICEF and Pure Earth [13]. The report also estimates that 35.5 million children have blood lead levels above five micrograms per deciliter (μg/dl), exceeding the CDC guidelines of below 3.5 micrograms per deciliter (μg/dl). Lead damages the brain and centra nervous system at high exposure levels, resulting in unconciousness, convulsions and even death. Children who survive severe lead poisoning may be left with intellectual disability and behavioral disorders. Exposure to lead lowers IQ and has major financial consequences [14].

### Global strategies for sustainable transport and decarbonization in energy‐economy models

Electric vehicles are appreciated globally for their smart mobility. The majority of electric vehicles are powered by the hydrocarbon combustion of fossil fuels; hence, transportation cost directly depends on the fossil fuel economy. Considering the price fluctuations of the fuel economy and greenhouse impact of CO_2_ emissions, new‐technology cars are primarily electronic. New policies, implementation mechanisms, and approaches are taken in many countries to create an inclusive environment for the acceptance of electronic vehicles. In Sri Lanka, import taxes on diesel‐based three‐wheelers have been increased to encourage the transition to environmentally friendly electric three‐wheelers. In the Philippines, the government has networked with the Land Bank of the Philippines and other financial conduits, such as rural banks, transport cooperatives, and multi‐cooperatives, to provide loan facilities to drivers of electric three‐wheelers. Going beyond financial assistance, Thailand has created a charging infrastructure [16].

### Reducing the burden by working on the framework gaps

The government can bring existing three‐wheeled motorized vehicles under the supervision of the BRTA. To strengthen the market, government bodies may provide vehicle standards. Necessary measures – adding an antilock brake system, separate route or limited area access permit, safety bells, etc. – can make electric three‐wheelers means of safe transportation, boost mobility, and save our environment and economy. More importantly, once it is integrated, it will establish a battery circular economy that will protect the environment from unregulated lead recycling by disposing of used batteries in a secure manner. Many countries are legislating electric three‐wheelers into the formal transport sector. It becomes easy to monitor and make policies when a transport mode is involved in the formal sector.

## CONCLUSION

In conclusion, from an environmental, socio‐economic, and legal perspective, the decision‐making bodies should keep electric three‐wheelers in consideration for convenience and to ensure a minimum carbon footprint. Electric three‐wheelers serve the purposes of all stakeholders. They provide a means of income for the physically disabled, while physically fit individuals can also drive them for their niche value. From a consumer point of view, they are quite cost‐effective. Rather than implementing a blanket ban, current and future research should be carried out with a focus on streamlining the industry, and it is recommended that BRTA introduce fitness documentation for electric three‐wheelers. Besides supporting partners in the national policy implementation process and promoting an evidence‐based approach, this commentary focuses on the sustainable policies and practices undertaken by other nations in a comparable environment.

## CONFLICT OF INTEREST

The authors declare no conflict of interest.

## Data Availability

All data related to this paper is available on request to the corresponding author.
